# A case of gastrolithiasis produced by a 5‐day diet

**DOI:** 10.1002/deo2.70012

**Published:** 2024-09-16

**Authors:** Akitoshi Hakoda, Kazuki Takayama, Shun Sasaki, Yosuke Mori, Hironiri Tanaka, Noriaki Sugawara, Taro Iwatsubo, Kazuhiro Ota, Hiroki Nishikawa

**Affiliations:** ^1^ Second Department of Internal Medicine Osaka Medical and Pharmaceutical University Osaka Japan

**Keywords:** anorexia, diet, endoscopy, gastrolith, stomach

## Abstract

A 55‐year‐old man with a history of distal gastrectomy was admitted to our hospital due to gastrointestinal bleeding from an anastomotic ulcer. After endoscopic hemostasis, his oral intake resumed after 1 day of fasting; however, he could not ingest food because of early satiety and nausea on the fifth day of oral intake resumption. Esophagogastroduodenoscopy was performed again to investigate the cause of anorexia and revealed a massive gastrolithiasis that was not observed in the previous esophagogastroduodenoscopy, which was diagnosed as the cause of his anorexia. Gastrolithiasis was treated with endoscopic removal the day after diagnosis, and the patient was discharged from the hospital after his symptoms resolved. Herein, we report the case of a patient with gastrolithiasis that developed and proliferated within 5 days.

## INTRODUCTION

Gastrolithiasis is a rare condition that develops in patients with delayed gastric emptying. The major symptoms of gastrolithiasis include early satiety and nausea, as reported previously.^1^ Similar symptoms have been observed in various diseases, and it is often difficult to determine when a diet causes the onset of gastrolithiasis. Gastrolithiasis is generally diagnosed through close examination after symptoms appear, and treatment options have been discussed in the literature.^2^, ^3^, ^4^, ^5^, ^6^ Here, we report a patient with gastrolithiasis that developed after a 5‐day diet. Notably, this is the first study to report on the duration of gastrolithiasis.

## CASE REPORT

**FIGURE 1:**
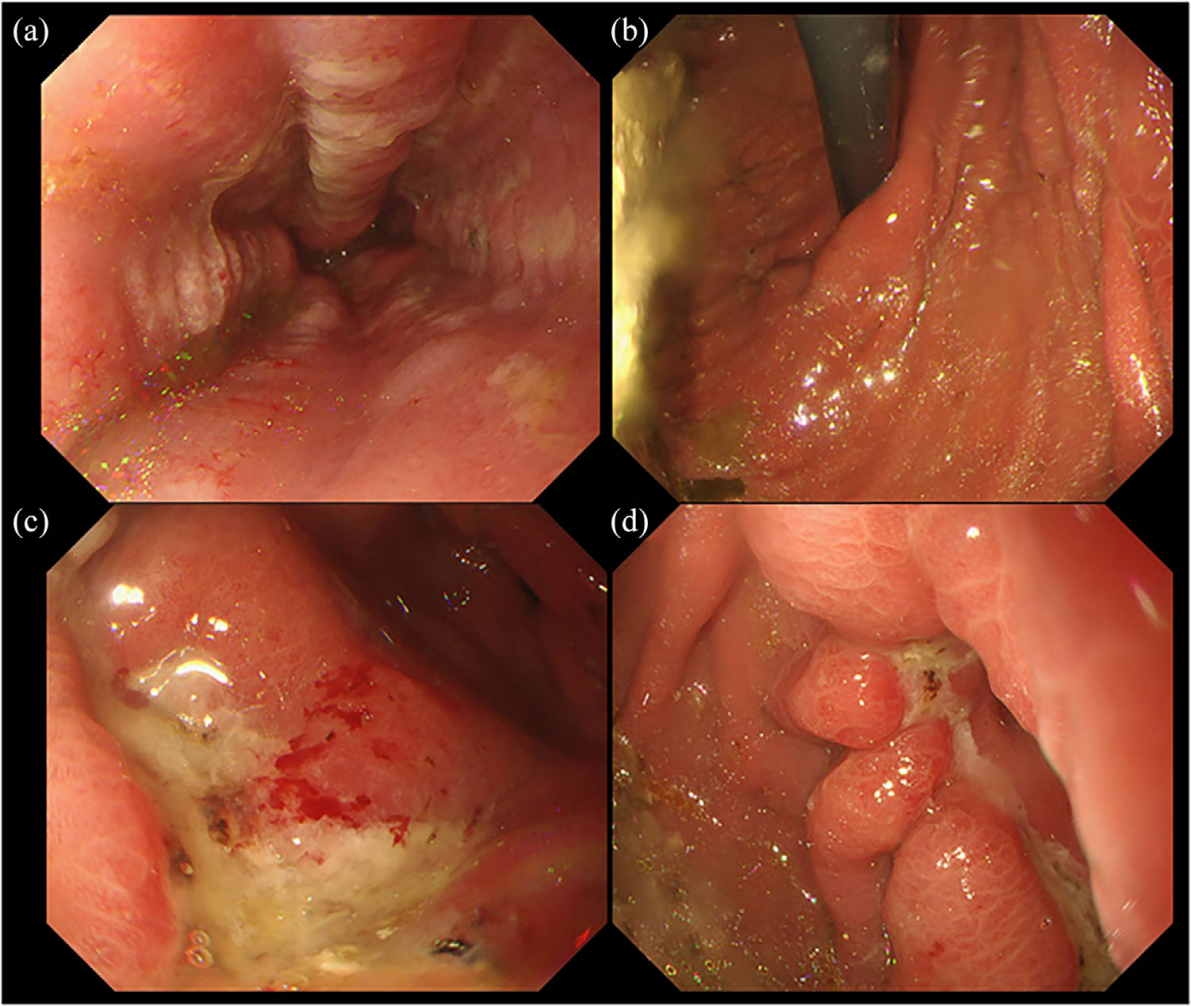
Gastroscopy findings on Day 0 for hematemesis scrutiny. (a) Abdominal esophagus. (b) Gastric fontanel and residue in the stomach. (c) Anastomotic ulcer of the stomach. (d) Anastomotic ulcer after hemostasis.

**FIGURE 2:**
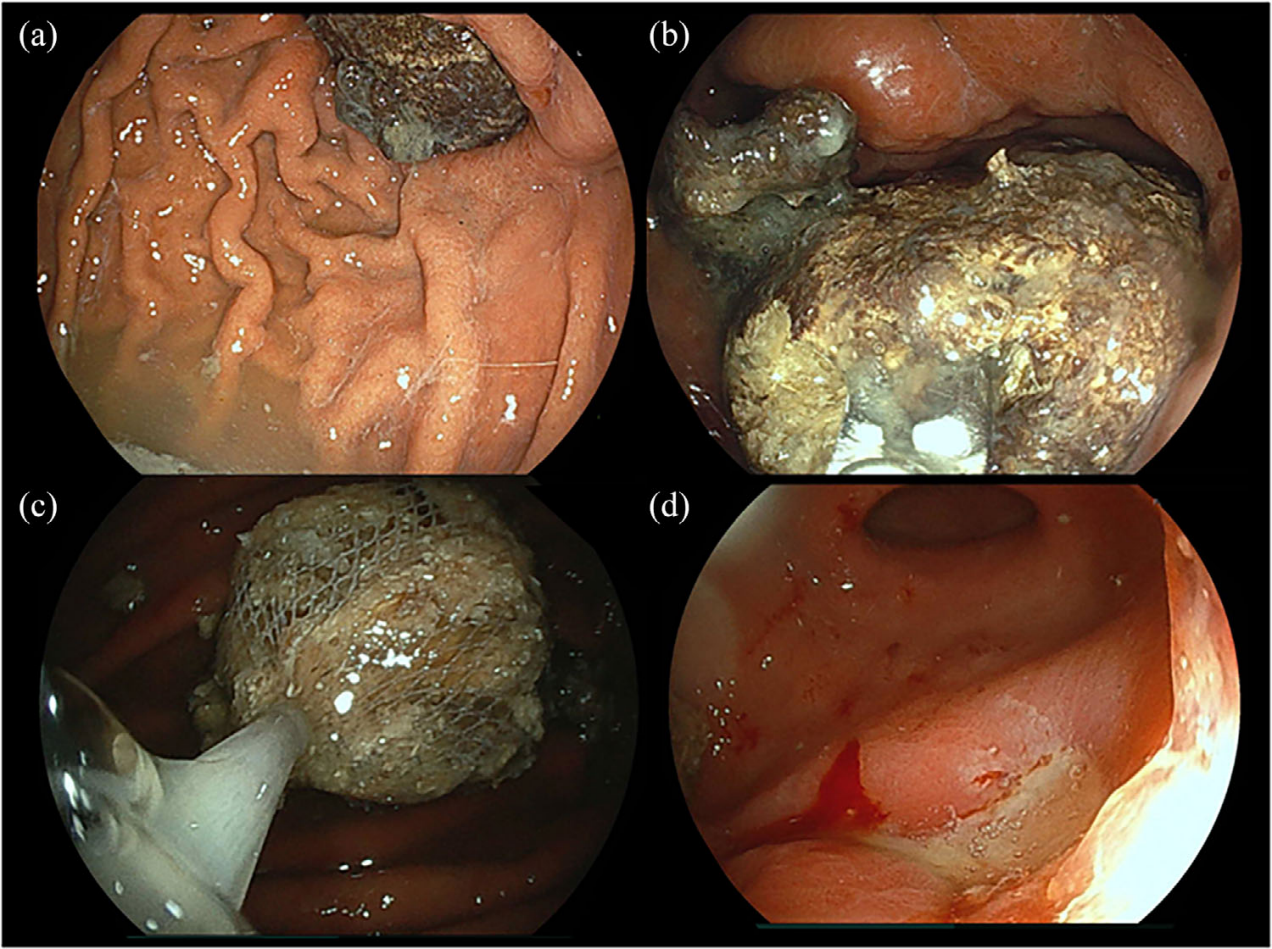
Gastroscopy findings on Days 8 and 9: Discovery of gastroliths. (a) Gastrolith (distant view image). (b) Gastrolith (close‐up image). (c) Images of gastrolith removal. (d) Image of anastomotic ulcer after gastric stone removal.

**FIGURE 3:**
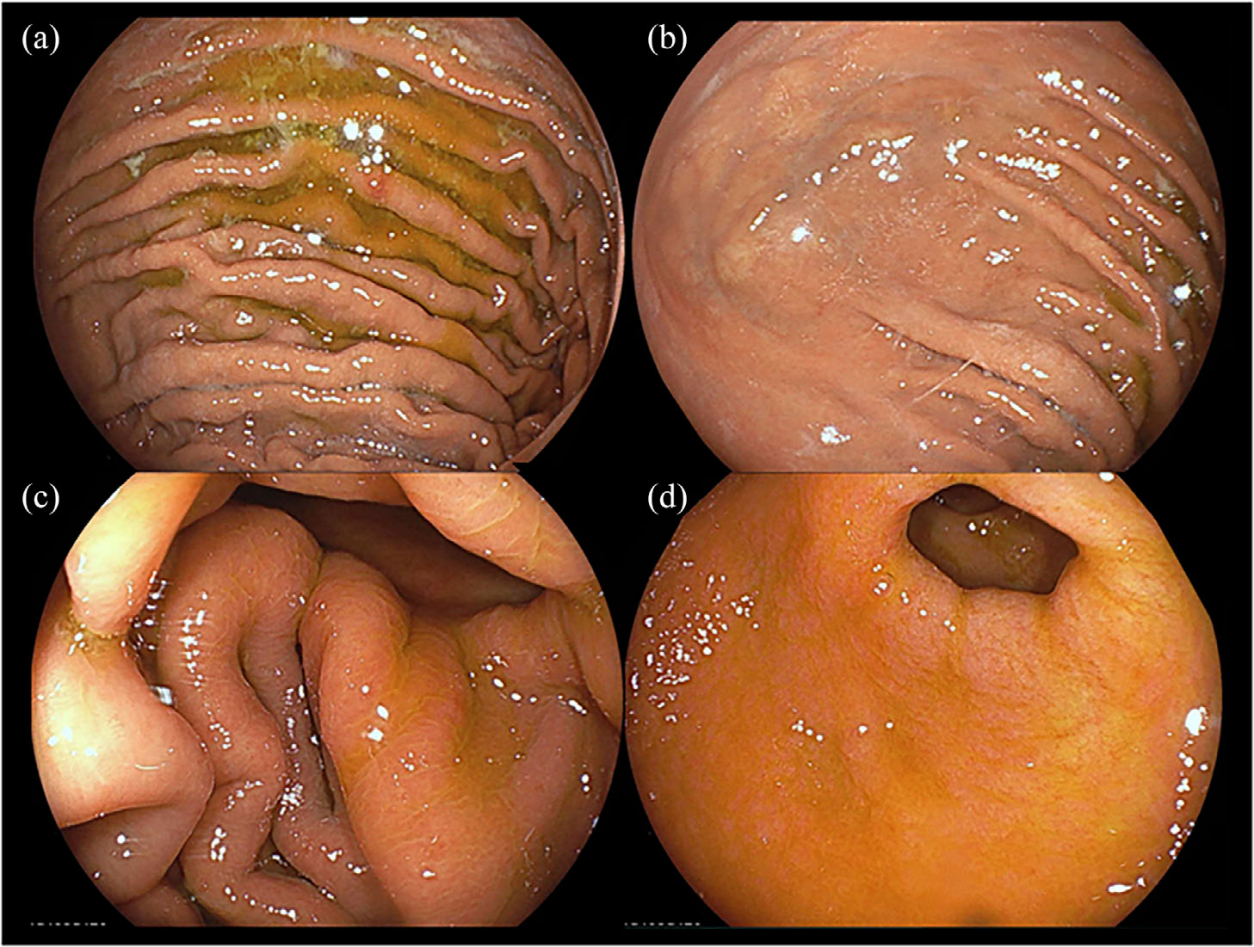
Gastroscopy findings 1 month after discharge. (a) Gastric body. (b) Gastric vault. (c) Postoperative anastomosis of the stomach. (d) Gastric hilum.

**FIGURE 4:**
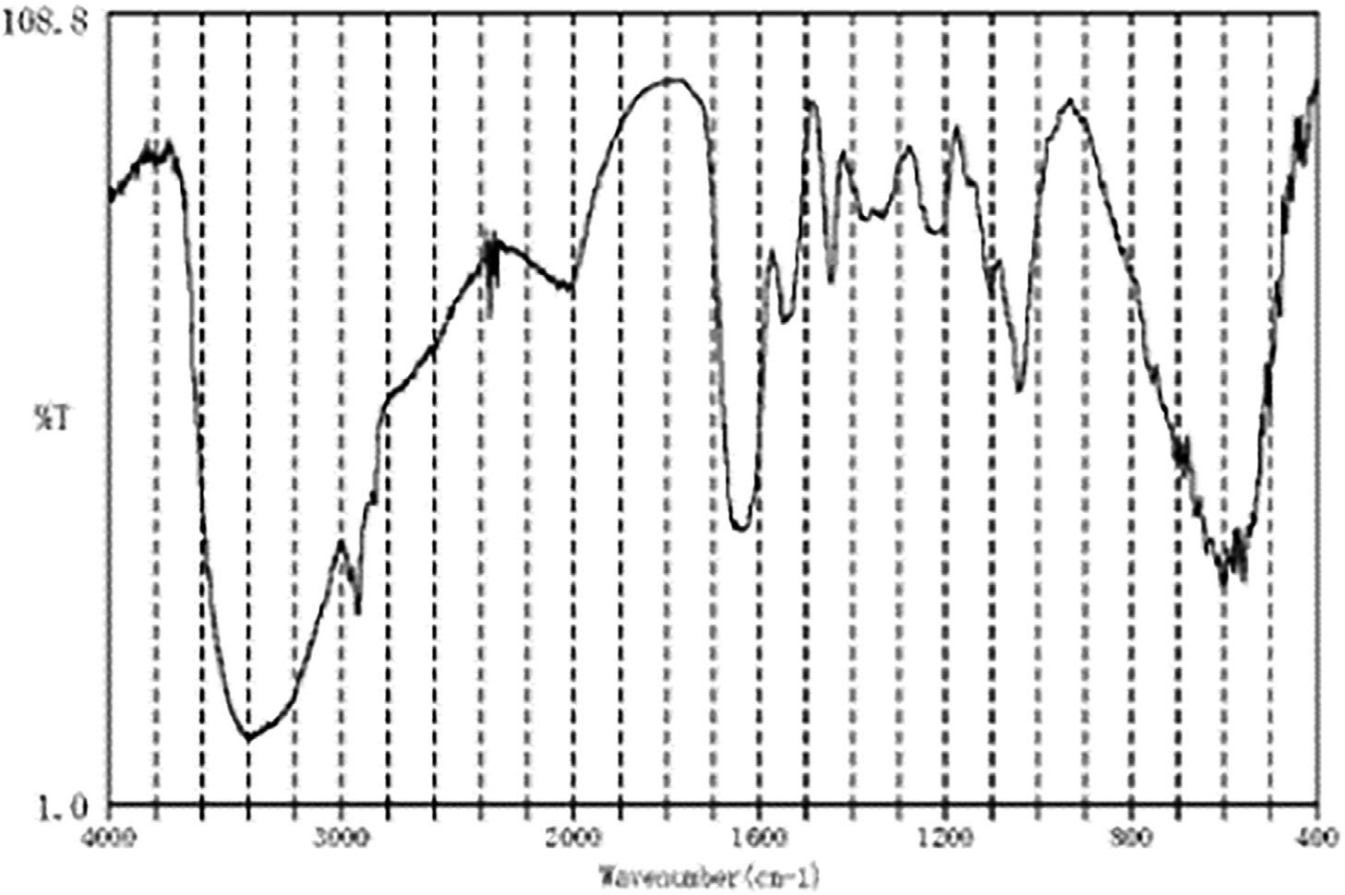
Component analysis of removed gastroliths.

A 55‐year‐old man with a history of pylorus‐preserving distal gastrectomy for the treatment of early gastric cancer 10 years prior was admitted to our hospital with the chief complaint of hematemesis (Day 0). Emergency esophagogastroduodenoscopy revealed an anastomotic ulcer with active bleeding, and endoscopic hemostasis was achieved using endoscopic forceps (Figure 1). A small amount of residue was observed (Figure 1b); however, no gastroliths were found later. Acid secretion inhibitors were used to treat gastric ulcers. An emergency endoscopy revealed a small amount of food residue in the remnant stomach with no signs of a gastrolith. The next day (Day 1), oral intake of a liquid diet was started, as there was no evidence of any suspected recurrence of upper gastrointestinal bleeding, such as a decrease in the hemoglobin level. On Day 3, the diet was modified to a slightly higher consistency; however, on Day 5, the patient started feeling early satiety and nausea and experienced difficulty eating. On Day 6, the patient could not eat, and intravenous infusion was resumed. Because of the persistent symptoms, an esophagogastroduodenoscopy was performed on Day 8 to follow up on the anastomotic ulcer and investigate the cause of anorexia. A large gastrolith (>50 mm) was observed on the distal side of the remnant stomach (Figure 2a and b). On the same day, attempts were made to crush and retrieve the gastrolith using forceps; however, gastric stone removal was challenging. On Day 9, another endoscopy was performed, and the gastrolith was crushed using a snare and collected through the mouth (Figure 2c,d). The patient was discharged on Day 12 without any epigastric symptoms or anorexia and underwent upper follow‐up gastrointestinal endoscopies at 1 and 6 months after discharge (Figure 3). No gastrolithiasis was noted in either follow‐up examination. Compositional analysis of the excised gastroliths revealed tannic acid as the major component (Figure 4).

## DISCUSSION

To the best of our knowledge, this is the first case report on the development and progression of gastrolithiasis. We consider this report valuable because it allowed us to evaluate the patient's situation endoscopically in real time, just before and after the onset of gastrolithiasis.

First, gastrolithiasis mainly relates to the consumption of foods that contain tannic acid and pectin, such as persimmon and jujube, in patients with a decreased expulsion capacity of the stomach.^7^ Tannic acid and pectin combine with proteins to form water‐insoluble tannic acid proteins deposited in the stomach. The patient was hospitalized and did not consume a diet high in tannic acid or pectin. Because of the postoperative stomach and the decreased excretory capacity of the stomach due to edema of the anastomotic ulcer, gastric calculus may have been caused by trace amounts of tannin contained in hospital food, such as green tea. The diet during the hospitalization period was reviewed and no tannin‐rich foods were found. Second, the patient remained bedridden and had little exercise during his hospital stay, which may have contributed to gastrointestinal emptying dysfunction and contributed to the development of gastrolithiasis. In addition, the vagus nerve was severed during a previous gastric surgery, contributing to decreased gastric excretory capacity. Third, hypo‐ and anacid conditions in the stomach reportedly cause gastrolithiasis.^6^ Since antisecretory agents were started on admission for treatment of anastomotic ulcers, the patient's stomach was expected to be in a low‐acid state.

The binding of water‐insoluble tannic acid to protein as a mechanism of gastrolithiasis is assumed to occur within a short period. However, it is thought that it takes time for the gastroliths to harden. In this patient, the gastrolith was large and soft, likely due to formation within a short period. The early detection of the gastrolith allowed it to be crushed and retrieved with an endoscopic snare or forceps.

Gastrolithiasis did not recur after hospital discharge due to the normalization of gastrointestinal peristalsis with the resumption of daily activities and the improvement in transit disturbance that had been associated with the anastomotic ulcer. Once the ulcer improved, the acid secretion inhibitor was also changed to a mucoprotective agent to prevent anastomotic ulcers while preventing low‐acid conditions in the stomach.

We learned two important lessons from this patient: (1) gastrolithiasis can occur within a short period, and (2) the early provision of food to patients with delayed gastric emptying can cause gastrolithiasis.

## CONFLICT OF INTERESTS STATEMENT

None.

## ETHICS STATEMENT

Not applicable.

## PATIENT CONSENT STATEMENT

Informed consent was obtained from the participant to publish this case report and associated images.
